# Use of a bio-electronic device comprising of targeted dual neuromodulation of the hepatic and celiac vagal branches demonstrated enhanced glycemic control in a type 2 diabetic rat model as well as in an Alloxan treated swine model

**DOI:** 10.3389/fnins.2022.1005932

**Published:** 2022-10-25

**Authors:** Jonathan J. Waataja, Raj K. Nihalani, Chris N. Honda, Charles J. Billington

**Affiliations:** ^1^ReShape Lifesciences Inc., San Clemente, CA, United States; ^2^Department of Neuroscience, University of Minnesota, Minneapolis, MN, United States; ^3^Division of Endocrinology and Diabetes, Department of Medicine, University of Minnesota, Minneapolis, MN, United States; ^4^Minnesota Veterans’ Administration Medical Center, Minneapolis, MN, United States

**Keywords:** bio-electronic, type 2 diabetes mellitus, neuromodulation, Vagus, diabetes

## Abstract

**Background:**

There is an unmet need for new type 2 diabetes treatments providing improved efficacy, durability and customized to improve patient’s compliance. Bio-electronic neuromodulation of Vagus nerve branches innervating organs that regulate plasma glucose, may be a method for treating type 2 diabetes. The pancreas has been shown to release insulin during Vagus stimulation. The hepatic vagal branch, innervating the liver, has been shown to decrease glucose release and decrease insulin resistance following ligation. However, standalone stimulation of the Vagus nerve has shown mixed results and Vagus nerve ligation has undesirable effects. Little is known; however, of the effect on plasma glucose with combined neuromodulation consisting of stimulation of the celiac branch innervating the pancreas with simultaneous high frequency alternating current (HFAC) blockade of the hepatic branch. This study tested the effects of this approach on increasing glycemic control in rat a model of type 2 diabetes and Alloxan treated swine.

**Materials and methods:**

Zucker obese (fatty) male rats (ZDF fa/fa) were used as a model of type 2 diabetes as well as glucose intolerant Alloxan treated swine. In ZDF rat experiments glycemic control was accessed with an intravenous glucose tolerance test during HFAC-induced hepatic branch block with concurrent celiac stimulation (HFAC + stimulation). In swine experiments glycemic control was accessed by an oral glucose tolerance test during HFAC + stimulation. Insulin measurements were taken prior to and following swine experiments giving insight into beta cell exhaustion. Histopathology was conducted to determine safety of HFAC + stimulation on Vagal branches.

**Results:**

Zucker rats demonstrated a significant improvement to an intravenous glucose tolerance test during HFAC + stimulation compared to sham. There was no significant difference from sham compared to hepatic vagotomy or celiac stimulation. In Alloxan treated swine, when subjected to HFAC + stimulation, there was a significant improvement in glycemic control as measured by an improvement on oral glucose tolerance tests and a decrease in fasting plasma glucose. Insulin responses were similar prior to and following HFAC + stimulation experiments. Histopathology demonstrated healthy swine Vagus nerves.

**Conclusion:**

Electrical blockade of the hepatic Vagus branch with simultaneous stimulation of the celiac Vagus branch may be a novel, adjustable and localized approach for a treatment of type 2 diabetes.

## Introduction

Despite medication, surgery and diet; type 2 diabetes mellitus (T2DM) remains challenging to effectively treat (Savoca and Miller, 2001; Nichols et al., 2007; Brethauer et al., 2013). For example, it has been estimated that 50% of type 2 diabetics do not take medications as prescribed (Nichols et al., 2007). Novel treatment options that are adjustable to patient’s compliance are needed. The Vagus nerve controls multiple organ systems that regulate blood glucose, such as the liver and the pancreas. Research into electrical neuromodulation of the Vagus nerve for a treatment of T2DM has shown mixed results. Chronic studies using stimulation or electrical conduction blockade of the Vagus nerve have demonstrated increased glycemic control; however, weight loss may have been a contributing factor (Shikora et al., 2016; Malbert et al., 2017) making it less suitable for many diabetics. Many stimulation studies of the Vagus nerve, or its branches, have failed to increase glycemic control (Daniel and Henderson, 1967; Frohman et al., 1967; Kaneto et al., 1967; Adrian et al., 1983; Berthoud et al., 1983; Ahren and Taborsky, 1986; Meyers et al., 2016), with only a few studies demonstrating enhanced glucose regulation (Yin et al., 2019). Since the Vagus nerve, and its branches, control multiple organ systems involved in blood glucose regulation, there may be necessity for electrical modulation of multiple branches to achieve optimal effectiveness to decrease blood glucose. This gave us the inspiration to test the effects of multisite/multi-frequency electrical neuromodulation of the Vagus nerve on glycemic control.

Many studies have shown that stimulation of Vagus nerve fibers innervating the pancreas causes an increase in plasma insulin, however, glucose levels are either unchanged or increased (Daniel and Henderson, 1967; Frohman et al., 1967; Kaneto et al., 1967; Adrian et al., 1983; Berthoud et al., 1983; Ahren and Taborsky, 1986). Vagus nerve stimulation-induced pancreatic secretion of glucagon may explain why glucose was not attenuated in these experiments (Adrian et al., 1983; Berthoud et al., 1983; Ahren and Taborsky, 1986). Ligation of neuronal fibers innervating the liver has been shown to affect plasma glucose (PG) possibly though disinhibition of vagal efferents innervating the pancreas (Lee and Miller, 1985), decreased hepatic sensitivity to glucagon (Xue et al., 2000) and/or decreased insulin resistance through attenuation of hepatic PPARα expression (Bernal-Mizrachi et al., 2007). However, ligation is non-reversible, the body may adapt to ligation over time and significant unwanted side effects may be associated with this technique such as negative changes in feeding behavior, increased hypoglycemic episodes, decreased liver regeneration and increased metastasis during liver cancer (Okazaki et al., 1993; Kiba, 2002; Donovan and Watts, 2014; Hiramoto et al., 2017). In one study hepatic vagotomy decreased insulin levels and increased PG (Okazaki et al., 1993).

**FIGURE 1:**
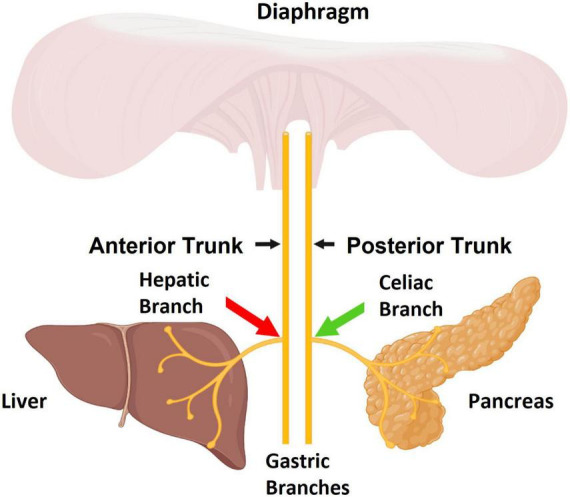
The sub-diaphragmatic Vagus nerve consists of two major trunks, the anterior and posterior. The pancreas is primarily innervated by the celiac branch which stems from the posterior Vagal trunk. The liver is innervated by the hepatic branch which stems from the anterior Vagal trunk. The aim of this study was to apply stimulation to celiac fibers (green arrow) and apply high frequency alternating current (HFAC)-induced blockade to hepatic fibers (red arrow). Figure created by www.biorender.com.

Combined stimulation of celiac fibers innervating the pancreas along with reversible electrical blockade of neuronal hepatic fibers innervating the liver is a new therapeutic concept that can be tested in an animal model of T2DM (sub-diaphragmatic Vagus nerve anatomy of interest and sites of neuromodulation are depicted in Figure 1). This dual procedure will also need to be compared to stand alone stimulation or standalone ligation in order to understand the new combined approach. To address this, we measured PG levels in an acute study following an intravenous glucose tolerance test (IVGTT) during stimulation of the celiac branch of the Vagus nerve while simultaneously using ligation or application of high frequency alternating current (HFAC), to the Vagus nerve hepatic branch in a Zucker obese (fatty) male rat (ZDF fa/fa) model of T2DM. High frequency alternating current is an established method of reversibly blocking conduction through nerve (Kilgore and Bhadra, 2013) and has been shown to block conduction in sub-diaphragmatic rat Vagus nerve (Waataja et al., 2011).

Studies were also conducted in control swine and glucose intolerant Alloxan treated swine. Swine have similar anatomy of metabolic organs and Vagus nerve size as humans, suggesting an appropriate human analog model to test Vagus nerve bio-electronic therapies. To obtain the parameters in which to block conduction through large diameter swine Vagus nerve *in vivo*, we performed isolated swine Vagus nerve electrophysiology experiments. These HFAC parameters are likely to translate to parameters required to induce conduction block *in vivo* by use of the same electrodes, similar impedances and similar contact with the Vagus nerves. We used the dual HFAC blockade and stimulation technique (HFAC + stimulation) in an *in vivo* study in Alloxan treated swine during an oral glucose tolerance test (OGTT). We hypothesized that this dual modulation will increase glycemic control in both Zucker rats and Alloxan treated swine. Dual modulation was compared to standalone celiac branch stimulation or standalone ligation of the hepatic branch. As far as we are aware, sending different patterns of electrical signals to two distinct branches of sub-diaphragmatic Vagus nerve for the treatment of a disease state has not been tested. The data of our study establishes the potential of using such a bio-electronic technique in the context of treating T2DM.

## Materials and methods

Rat experiments were approved by the Institutional Animal Care and Use Committee at the University of Minnesota. Swine experiments were approved by both the Institutional Animal Care and Use Committee at North American Science Associates, Inc. (Brooklyn Park, MN, USA) as well as the Institutional Animal Care and Use Committee at the University of Minnesota.

### Rat experiments

**FIGURE 2:**
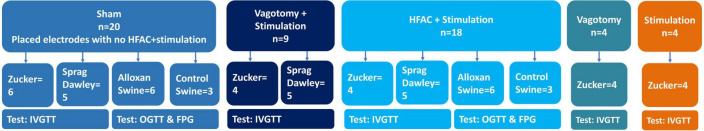
Depiction of experimental groups and animal numbers/experimental condition.

Male Zucker obese (fatty) rats (ZDF fa/fa) (ZDF rats) (63 days old), or adult male Sprague Dawley control rats, were given food *ad libitum* (Purina #5008 for ZDF rats and Envigo 2918 for Sprague Dawley) except for an 18 h fast prior to IVGTT experiments. Rats were anesthetized with an intraperitoneal (IP) injection of sodium pentobarbital (40–50 mg/kg). Next, rats were placed on a heating blanket and the right jugular vein was cannulated. The depth of anesthesia was assessed periodically by testing a paw withdrawal reflex. If a reflex was observed a maintenance dose (5 mg/kg) of pentobarbital was administered IV. Next, the abdominal cavity was opened, and the liver retracted. The hepatic branch of the anterior sub-diaphragmatic Vagus nerve and the celiac branch of the posterior sub-diaphragmatic Vagus nerve were isolated and separated from the esophagus. There were 5 experimental conditions (a visual depiction of experimental groups can be seen in Figure 2): (1) sham operation (nerve isolation only, ZDF *n* = 6, Sprague Dawley *n* = 5), (2) vagotomy + stimulation (positive control, ZDF *n* = 4, Sprague Dawley *n* = 5), (3) HFAC + stimulation (ZDF *n* = 4, Sprague Dawley *n* = 5), (4) vagotomy alone (ZDF *n* = 4), and (5) stimulation alone (ZDF *n* = 4). In the vagotomy + stimulation group the hepatic branch was ligated, and the celiac branch was stimulated at 1 Hz. In the HFAC + stimulation group, a 5,000 Hz alternating current signal was applied to the hepatic branch, and the celiac branch was stimulated at 1 Hz. In the vagotomy alone group the hepatic branch was ligated. In the stimulation alone group the celiac branch was stimulated at 1 Hz and the hepatic branch remained intact.

Stimulation and HFAC parameters consisted of the following: the celiac branch was suspended on bipolar platinum/iridium wires (0.01 inch diameter). A monophasic negative square wave with a pulse width of 4 millisecond was generated by a grass s44 stimulator (Grass Medical Instruments, Quincy, MA, USA) which drove a stimulus isolation unit (Model A360, World Precision Instruments, Sarasota, FL, USA) at 1 Hz. The pulse amplitude was 8 mA. For delivery of HFAC, the hepatic branch was suspended on bipolar platinum/iridium ribbon wires (0.02 inch thickness; 0.05 inch width). The electrode made a 180° contact with the nerve. The current amplitude was 8 mA.

One hour following all procedures (except for 15 min following the HFAC + stimulation procedure) a blood sample was taken from a cut end of the rat’s tail. An AlphaTrak (Abbott Laboratories, North Chicago, IL, USA) blood glucose monitor was used to measure blood glucose concentrations (mg/dL). Typical fasting glucose (18 h fast) for ZDF rats was 200 mg/dL and 150 mg/dL for Sprague Dawley control rats. Next, an IVGTT was performed. The IVGTT consisted of an IV injection into the port of a 0.5 g/kg dose of glucose made up in 0.9% saline with a 20% weight/volume concentration (Nagase et al., 1993). Blood glucose was then sampled for 30 min following the glucose injection. Stimulation and/or delivery of HFAC were maintained during the IVGTT. In some cases, a subsequent IVGTT was administered in the sham group and 15 min following the cessation of HFAC + stimulation signals.

### High frequency alternating current

**FIGURE 3:**
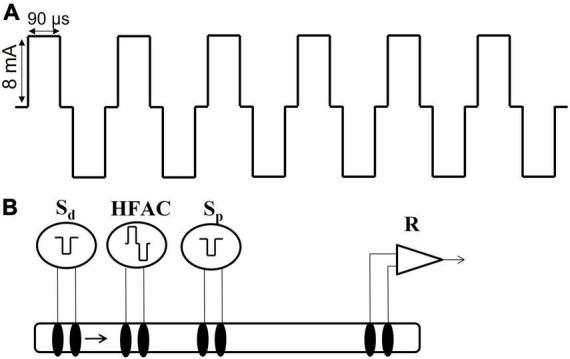
High frequency alternating current waveform and electrode configuration for the isolated nerve electrophysiology studies. **(A)** The high frequency alternating current (HFAC) was a 5,000 Hz signal with 90 us square wave pulses with a 10 us shorting period between the charge and recharge phases and delivered at 8 mA. This HFAC signal was used in the isolated nerve electrophysiology experiments as well as the *in vivo* rat and swine experiments. **(B)** The electrode configuration used in the isolated vagus nerve electrophysiology experiments consisted of a distal stimulation electrode (S_d_), an electrode delivering HFAC, a proximal control stimulation electrode (S_p_) and a recording electrode (R). The segment of nerve between the proximal stimulation and the recording electrodes was submerged in oxygenated SIF. The proximal stimulation electrode was used to test for HFAC-induced excitation which may produce fatigue following cessation of the HFAC signal; as opposed to a prolonged depolarization block at the site of the HFAC electrode.

A Viking Model 2002 neuroregulator (ReShape Lifesciences Inc., San Clemente, CA, USA) was used to generate a 5,000 Hz signal in all experiments involving HFAC. The signal consisted of a bi-phasic constant current square waveform consisting of a charge component and a recharge component (Figure 3A). The charge and recharge waveform components were of opposite polarity and were generated from the same current source so the current in each component was matched. The output of the pulse generator was not capacitively coupled but rather employed a proprietary method for charge balance. Shorting periods (10 μs) were incorporated as part of the duty cycle of each of the charge and recharge waveform components. During these shorting periods, the electrodes were short-circuited together to remove any charge remaining after application of the waveform. The pulse generator has been measured for direct current (DC) and consistently met a <1 μA leakage current specification; typically < 50 nA.

### *In vivo* swine experiments

Adult Yucatan swine (∼45 kg, *n* = 6, unless otherwise specified) were allowed to acclimate for 7 days following shipment from Sinclair Bio Resources (Auxvasse, MO, USA). In 6 swine a proprietary titrated dose of Alloxan was administered at Sinclair Bio Resources (Auxvasse, MO, USA) to the swine *via* an IV injection 8 weeks prior to shipment. The swine were monitored and fed *ad libitum* for 24 h following Alloxan treatment to prevent any possible hypoglycemia due to release of insulin into the blood due to beta cell death. The swine were not insulin dependent. Swine were offered food twice per day (Teklad 7200, Envigo, for the non-diabetic swine and CU Sinclair S-9 Ration, Sinclair Bio Resources, for the Alloxan treated swine) except for an 18 h fast prior to glucose challenges. Intravenous glucose tolerance tests (IVGTT) experiments, used to test for glucose intolerance in Alloxan treated swine, consisted of an IV injected of 600 mg/kg glucose and blood sampled with a glucometer (One Touch Ultra, LifeScan, Malvern, PA, USA).

Swine were trained to drink 100 mL of diet Gatorade delivered through a syringe as well as to wear a jacket to house mobile charging units for future charging sessions. The oral glucose tolerance tests (OGTTs) consisted of oral consumption of 75 g of glucose dissolved in 100 mL of diet Gatorade. Oral glucose tolerance tests are widely used to measure glycemic control (Khan et al., 2019; Kuo et al., 2021) including in swine (Manell et al., 2016, 2021). An IV port was placed in the jugular vein. Insulin was measured using a Millipore Sigma RIA kit (#HI-14K, Burlington, MA, USA).

For surgical implantation of the Viking neuroregulators (ReShape Lifesciences Inc., San Clemente, CA, USA) (2) and Viking Model 2200-47E leads (ReShape Lifesciences Inc., San Clemente, CA, USA) (4), swine were anesthetized with Telazol/Xylazine given IM at a dose of 6 and 1 mg/kg Xylazine. Animals were intubated and maintained on isoflurane inhalant anesthetic to effect (1.0–2.0%). Two Viking leads with platinum iridium cuff electrodes, which made a 180° contact with the nerve, were placed on the anterior sub-diaphragmatic vagal trunk cranial to the hepatic branching point (referred to the hepatic branch in swine experiments) and sutured onto the esophagus to deliver HFAC (5,000 Hz, 8 mA current amplitude). A second pair of identical cuff electrodes were placed on the posterior sub-diaphragmatic vagal trunk cranial to the celiac branching point (referred to as the celiac branch in swine experiments) and sutured onto the esophagus to deliver a bi-phasic charge balanced pulse at 1 Hz (4 ms pulse width and 8 mA current amplitude). Each lead had a wing suture tab between the electrode tip and the connection to the neuroregulator. The wing was sutured to the stomach as a strain relief.

**FIGURE 4:**
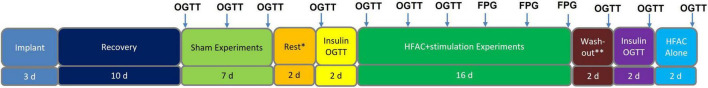
Study timeline for Alloxan treated swine experiments. Each arrow indicates an experiment. Each experiment was conducted in 1 day. There were 2 days of separation between Sham oral glucose tolerance tests (OGTTs) as well as 2 days of separation between high frequency alternating current (HFAC) + stimulation OGTTs and fasting plasma glucose (FPG) experiments. In Sham and OGTT experiments FPG was the baseline blood sample (swine were fasted 18 h prior to all experiments). *Rest involves recovery from OGTTs. ^**^Depicts rest period prior to washout OGTT.

**FIGURE 5:**
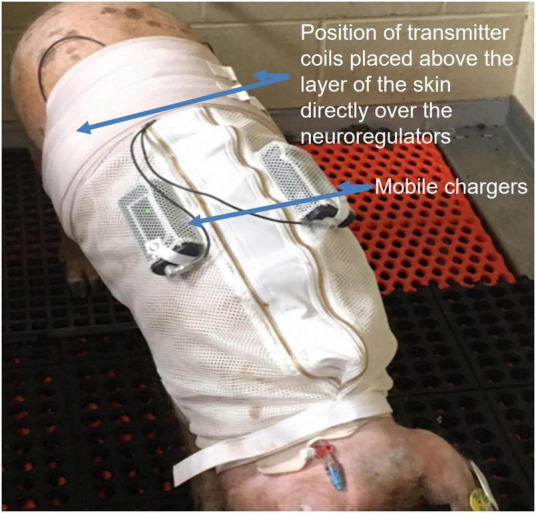
Alloxan treated Yucatan swine wore a specially designed jacket to house two of the ReShape Lifesciences Inc. Viking mobile chargers. Transmitter coils above the layer of the skin were connected to the mobile chargers to charge the implanted neuroregulators with a RF signal between experiments. Settings for stimulation and high frequency alternating current (HFAC) parameters were also programmed into the neuroregulators using a laptop computer and application software *via* the mobile chargers and transmitter coils. Swine were trained for 7 days to wear the jacket. During the charging sessions the swine were not restrained and there was no apparent stress to the animals.

Leads were tunneled to 2 Viking neuroregulators in a subcutaneous pocket above the ribcage on either side of the swine. The swine were allowed to recover for 10 days following implant before OGTTs were performed (timeline of experiments can be seen in Figure 4). To charge the neuroregulators, following HFAC + stimulation experiments, a coil was positioned over the neuroregulator above the layer of the skin (Figure 5). The coil was then connected to a Viking mobile charger and delivered a 6.78 MHz radio frequency signal to the implanted neuroregulator. Sham conditions consisted of the devices implanted but not delivering HFAC or stimulation signals.

### Isolated swine Vagus nerve electrophysiology

Swine Vagus nerves were harvested following euthanization. Next, the esophagus, with Vagus nerve trunks attached, was extracted from the level of the stomach to below the level of the heart. The block of tissue was then placed in ice cold oxygenated synthetic interstitial fluid [SIF; containing (in mM) 123 NaCl, 3.5 KCl, 0.7 MgSO4, 2.0 CaCl2, 9.5 Na gluconate, 1.7 NaH2PO4, 5.5 glucose, 7.5 sucrose, and 10 HEPES; pH 7.45] (Koltzenburg et al., 1997). Next, both anterior and posterior nerve trunks were dissected from the esophagus at the sub-diaphragmatic gastric branches to a level below the heart and placed in ice cold oxygenated SIF.

Excised nerves were positioned in a recording chamber on four sets of bipolar hook electrodes and suspended in mineral oil. The recording chamber was suspended inside a hot water bath held at 34°C. The electrode arrangement was similar to that in Waataja et al. (2011) with an electrode delivering HFAC positioned between stimulation (“distal” electrode) and a recording electrode. The distal stimulation electrode was positioned just above the gastric branches, below the level of the diaphragm as well as the electrodes delivering HFAC (with 2 mm separation between the HFAC electrodes). The recording electrode was placed at the opposite rostral end of the nerve segment. A “proximal” control stimulating electrode was positioned between the blocking electrode and the recording electrode (Figure 3B). The stimulation and recording electrodes consisted of pairs of platinum/iridium and Ag/AgCl wire (0.01–0.015 inch diameter), respectively. The electrode delivering HFAC consisted of a pair of platinum–iridium ribbon wires (width of 1.4 mm) in a hook configuration which cradled the nerve (180 degrees of contact); similar to the *in vivo* swine experiments. The nerve made contact with a layer of oxygenated SIF below the mineral oil, between the proximal stimulation and recording electrodes, which helped supply oxygen/nutrients and provided a grounding path. Temperature measurements were taken inside the recording chamber to assure the nerve was exposed to a constant temperature of 34°C.

The recording chamber consisted of an inner and outer chamber. The outer chamber contained a thermostatically controlled heating element submerged in water. The outer chamber housed an inner chamber holding the Vagus nerve in mineral oil with the underlying layer of oxygenated SIF. The Vagus nerve was electrically activated through the stimulation electrodes with monophasic (negative) pulses generated by a constant current stimulus isolation unit (A385, World Precision Instruments, Sarasota, FL, USA) driven by a pulse generator (Isostim A320, World Precision Instruments, Sarasota, FL, USA) at 1 Hz. Typical stimulus durations were 0.1–0.5 ms and amplitudes 0.5–4.5 mA. The methodology for the proximal and distal stimulation was the same except the proximal electrode was activated 200 ms following the activation of the distal stimulation electrode. Stimulus-evoked nerve signals were led from recording electrodes to the headstage of a differential amplifier (DAM 80, 10,000X gain, typical bandpass of 10 Hz to 3 kHz, World Precision Instruments, Sarasota, FL, USA) and referenced to a Ag/AgCl pellet in the underlying SIF. The resulting signal was led in parallel to an oscilloscope and a data acquisition system (Power 1401 with Spike 2, Cambridge Electronic Design, Cambridge, UK).

The 5,000 Hz signal was applied for 1 min. Baseline compound action potentials (CAPs) were recorded 1 min prior to the application of the 5,000 Hz HFAC signal at a rate of 1 Hz. Compound action potential amplitude was normalized to average baseline values. Following cessation of 5,000 Hz a CAP was elicited within 1 s and was defined as the degree of block (or “CAP Amplitude”) and graphed on a current-effect curve.

### Nerve histopathology

The collected fixed Vagus nerves were sectioned where lead cuff was located following HFAC + stimulation experiments. The tissue sections were trimmed, paraffin embedded, sectioned at ∼5 microns (cuff placement site), mounted to slides and stained with hematoxylin and eosin (H&E) for microscopic evaluation. Qualitative and semi-quantitative histopathologic evaluation of the local tissue effects and the inflammatory response was conducted. Biological response parameters were analyzed for local effects of implantation based upon ISO standards 10993-6 Biological Evaluation of Medical Devices. Tests for local effects after implantation including inflammatory cells, fibroblasts, neovascularization, fibrosis, fatty infiltrates, necrosis, and mineralization. Tissue ingrowth, foreign debris, and axonal damage were also evaluated. Additionally, test site encapsulation thickness was estimated.

### Analysis

To decrease the effective variability in fasting plasma glucose (FPG) between animals and to make comparisons between species, changes in glucose were normalized to baseline glucose; similar to Sgambato et al. (1986). Baseline glucose was measured 5 min prior to the IVGTT in rats and 10 min prior to the OGTT in swine. Percent change in glucose concentration was calculated using the following equation:

% Change = [(glucose concentration at time × – Baseline glucose concentration)/(Baseline glucose concentration)] × 100

The glucose response was quantified by calculating the area under the curve [AUC, % change in glucose concentration × time = area units (AU)] (Berthoud et al., 1983; Lee and Miller, 1985; Lamont and Drucker, 2008). The area between a line connecting two subsequent data points and the x-axis was calculated as one segment. The total number of segments following the glucose challenge was then summated. The AUCs were averaged for 3 Sham experiments and AUCs were averaged for 3 HFAC + stimulation experiments. For rat experiments, comparisons between the condition tested and sham consisted of a Student’s *t*-test with a nominal alpha level of 0.05 as considered significant. There were no adjustments for multiplicity. For comparisons of multiple means a One-way ANOVA was utilized. All data are presented as mean ± SEM.

## Results

### High frequency alternating current with concurrent stimulation improved performance on an intravenous glucose tolerance test in male Zucker obese (fatty) rats

The ZDF rat is a well-established rodent model of T2DM (Friedman et al., 1991; Sparks et al., 1998; Corsetti et al., 2000; Schmidt et al., 2003; Reinwald et al., 2009). ZDF rats are homozygous for a non-functional leptin receptor which causes obesity and insulin resistance (Chen and Wang, 2005). Pancreatic ß-cells have also been shown to fail to respond to glucose in these rats (Chen and Wang, 2005). This model was used to test our hypothesis that HFAC applied to the hepatic branch of the Vagus nerve with concurrent celiac branch stimulation will reversibly increase glycemic control in an animal model of T2DM. To access glycemic control an IVGTT was chosen over an oral or intra-peritoneal glucose challenge because the rat was anesthetized with its abdominal cavity exposed.

**FIGURE 6:**
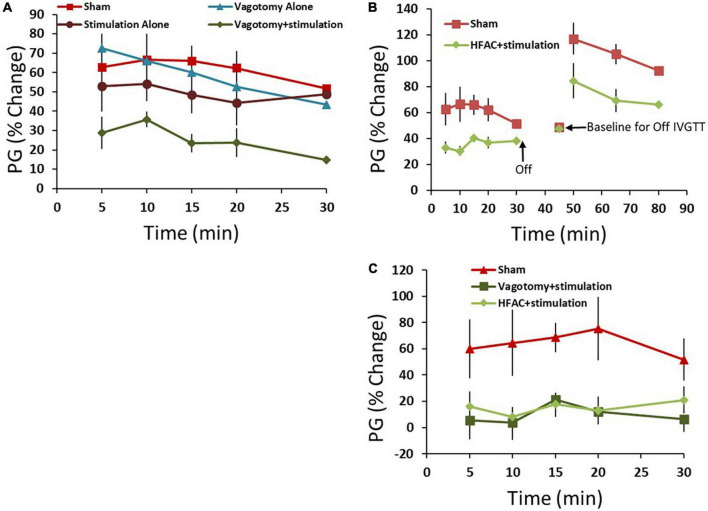
High frequency alternating current applied to the hepatic branch of the vagus nerve with simultaneous stimulation of the celiac branch of the vagus nerve reversibly decreased plasma glucose (PG) following an intravenous glucose tolerance test (IVGTT) in control and Zucker obese (fatty) male (ZDF) rats compared to sham. **(A)** As a positive control the hepatic nerve was ligated [in place of application of high frequency alternating current (HFAC)] with concurrent stimulation of the celiac nerve. This produced increased glycemic control compared to sham following an IVGTT. Standalone celiac nerve stimulation or hepatic nerve ligation had the same response to the IVGTT as sham. **(B)** Similar to vagotomy + stimulation, the application of HFAC + stimulation increased glycemic control following the IVGTT compared to sham. A subsequent IVGTT 15 min following the cessation of HFAC + stimulation demonstrated decreased glycemic control; suggesting reversibility. **(C)** Control Sprague Dawley rats also demonstrated increased glycemic control with HFAC + stimulation and vagotomy + stimulation. Error bars are SEM.

First, control experiments were performed which consisted of 4 conditions: a sham operation, a vagotomy + stimulation positive control vagotomy and stimulation (Figure 2). All comparisons were made only between the experimental group and sham and statistics included a Student’s *t*-test with no adjustments for multiplicity. Multiple comparisons between groups were not made. One hour following these procedures an IVGTT was administered. Plasma glucose (PG) increased by an average of 63 ± 12% 5 min following the glucose injection in the sham group and remained elevated for a half hour with a partial recovery (Figure 6A, AUC = 1543 ± 257 AU). For the hepatic vagotomy or stimulation groups there were no significant difference in the AUC compared to sham following the challenge (Figure 6A, vagotomy AUC = 1425 ± 157 AU, stimulation AUC = 1220 ± 250 AU). However, there was a significant decrease in AUC compared to sham in the vagotomy + stimulation group following the challenge (Figure 6A, AUC = 618 ± 111 AU, *p* < 0.01).

To test if HFAC applied to the hepatic branch with concurrent celiac stimulation mimicked the increased glycemic control as in the vagotomy + stimulation group, HFAC and stimulation were applied 15 min prior to and during a 30 min IVGTT. Following the IVGTT there was a significant decrease in AUC compared to sham (Figure 6B, AUC = 898 ± 68, *p* < 0.05). Fifteen mins following the cessation of HFAC + stimulation a second glucose injection induced a large increase in AUC which was non-significant, albeit slightly attenuated, to a subsequent glucose injection in the sham group (Figure 6B). This suggests a functional recovery following cessation of HFAC + stimulation.

Experiments in control Sprague Dawley rats demonstrated a similar and significant pattern as in ZDF rats. In the sham group glucose increased by 60 ± 22% following administration of glucose with a partial recovery at 30 min. When the celiac branch was stimulated with either a concurrent hepatic ligation or concurrent delivery of 5,000 Hz there was a significant decrease in glucose following the challenge compared to sham (Figure 6C, sham = 1704 ± 553 AU, vagotomy + stimulation AUC = 202 ± 322 AU *p* < 0.05, HFAC + stimulation AUC = 418 ± 140 AU, *p* < 0.05). In all conditions for both ZDF and Sprague Dawley rats glucose remained steady for the treatment period prior to the IVGTT.

### High frequency alternating current blocked conduction through porcine Vagus nerve

A 5,000 Hz HFAC signal has been shown to reversibly block conduction through sub-diaphragmatic rat Vagus nerve and low frequency sub-diaphragmatic Vagus nerve monophasic stimulation parameters have been established (Waataja et al., 2011). However, little is known of the effects of application of 5,000 Hz HFAC on larger sub-diaphragmatic swine nerves and optimal parameters for bi-phasic stimulation. To test current amplitudes required to block and stimulate the sub-diaphragmatic swine Vagus nerve, we observed electrically elicited compound action potential (CAPs) (*n* = 5). The isolated nerve was suspended on 4 hook electrodes; a distal stimulation electrode, an electrode delivering HFAC, a control proximal stimulation electrode and a recording electrode (Figure 3B).

**FIGURE 7:**
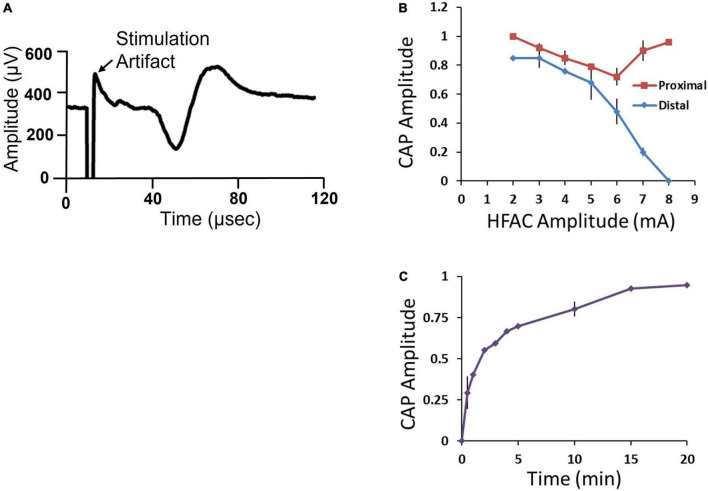
Isolated swine sub-diaphragmatic vagus nerve electrophysiology demonstrated conduction block and recovery at a high frequency alternating current (HFAC) amplitude of 8 mA. **(A)** Representative compound action potential (CAP) elicited by electrical stimulation at the level below the diaphragm and recorded at a segment below the heart. The length of the isolated vagus nerve was approximately 35 mm. **(B)** Current-effect curve of CAP amplitude elicited from the proximal and distal stimulation electrodes immediately following the cessation of HFAC. CAP amplitude was normalized to baseline. **(C)** Recovery of CAP amplitude following full block. Error bars are SEM.

The distal and proximal stimulation electrodes both elicited a CAP (Figure 7A). However, when HFAC was applied the distal CAP amplitude decreased in a current dependent manner with consistent full block at 8 mA (Figure 7B). The proximal electrode was used as a control to test for repetitive firing of action potentials which has been shown to occur with the application of HFAC (Kilgore and Bhadra, 2013). If the HFAC elicited anti- and ortho-dromic action potentials there would be collision blocks with action potentials elicited by the stimulation electrodes decreasing the amplitude of the CAPs. There was a decrease in the CAP produced by the proximal electrode in a current dependent manner at HFAC amplitudes less than 6 mA which peaked at an average of a 28% decrease (Figure 7B). However, as the HFAC current amplitude was increased the proximal CAP increased to baseline values with only an average of a 4% decrease at 8 mA. Following cessation of HFAC the distal CAP amplitude returned to 90% of its baseline amplitude within 15 min, and ≥95% at 20 min, on average (Figure 7C). The conduction block that persisted following the cessation of HFAC has been reported previously and termed a “carry-over” effect (Waataja et al., 2011; Cuellar et al., 2012; Kilgore and Bhadra, 2013).

Stimulation of the sub-diaphragmatic swine Vagus nerve was tested using a charge balanced bi-phasic square wave generated by the Viking neuroregulator. Maximal CAP amplitudes consistently occurred at current amplitudes of 8 mA and pulse widths of 4 ms.

### High frequency alternating current with simultaneous stimulation increased glycemic control in Alloxan treated swine

**FIGURE 8:**
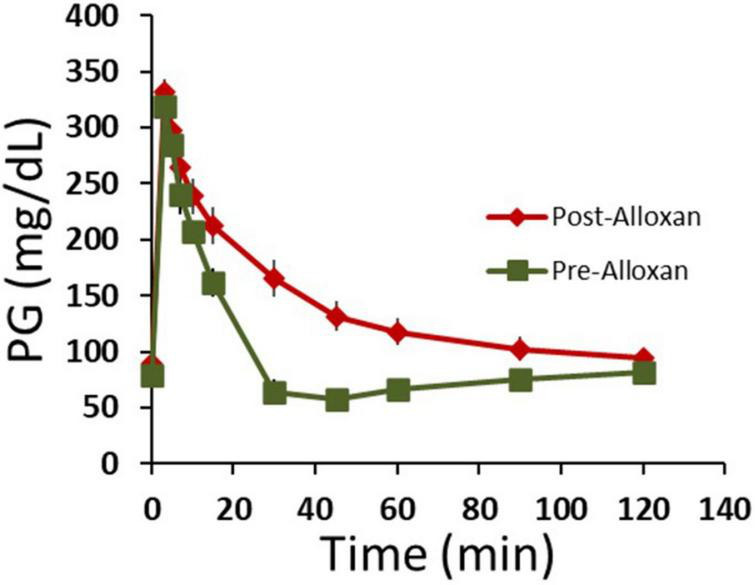
Following Alloxan treatment swine demonstrated glucose intolerance. Graph depicts plasma glucose levels during an intravenous glucose tolerance test (IVGTT) prior to and following the administration of Alloxan to the swine. *n* = 6 swine. Error bars are SEM.

We next tested HFAC + stimulation in an Alloxan treated swine. By a proprietary method at Sinclair Bio Resources (Auxvasse, MO, USA), a titrated dose of Alloxan-induced partial ablation of beta cells. Following Alloxan treatment, swine had decreased glycemic control but were not insulin dependent. An IVGTT was conducted prior to, and following, the Alloxan treatment which demonstrated significantly increase in AUC following Alloxan (Figure 8, pre-Alloxan AUC = 3237 ± 362 AU, post-Alloxan AUC = 7230 ± 483 AU, *p* < 0.001, Student’s *t*-test). Following recovery from IVGTTs PG was 113 ± 9 mg/dL. Next, two Viking cuff electrodes were implanted on the vagal trunks as described in Section “Materials and methods.” The anode and cathode electrodes delivering HFAC and stimulation were the same dimensions, configuration, separation, and impedance (typically 1,000 ohms) as used in the isolated swine Vagus nerve electrophysiology study.

**FIGURE 9:**
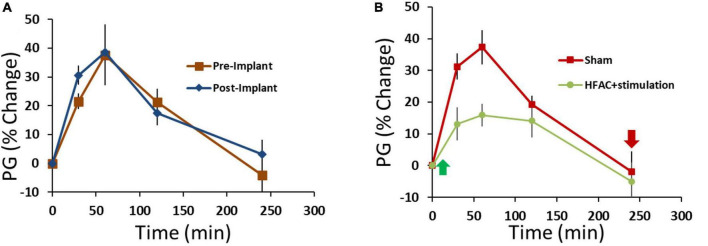
Application of high frequency alternating current (HFAC) to the hepatic branch of the vagus nerve with simultaneous stimulation of the celiac branch of the vagus nerve increased performance on an oral glucose tolerance test (OGTT) compared to pre-device initiation OGTTs in Alloxan treated swine. **(A)** Results from an OGTT conducted prior to implant of the device and following the implant of device were similar; suggesting that the implant itself did not change glycemic control. *n* = 3 swine. **(B)** Application of HFAC + stimulation 5 min following the initiation of the OGTT significantly improved performance on the OGTT in Alloxan treated swine *n* = 6 swine. Green arrow depicts when HFAC + stimulation was turned on and red arrow depicts when HFAC + stimulation was tuned off. *n* = 6 swine. Error bars are SEM.

Following 10 days of recovery from surgery 3 OGTTs were conducted (2 days separation between OGTTs, Figure 4) with the devices off (sham OGTT). The results from the OGTTs were consistent, no significant difference, and similar to the pre-implant OGTTs (Figure 9A). Following 2 days of rest, an OGTT was conducted, and insulin measured to test the pre-device initiation insulin response. Following a 2-day rest, 3 HFAC + stimulation OGTT experiments were conducted (2 days separation between OGTTs, Figure 4). The HFAC + stimulation on time was 3 h and 55 min occurring during the OGTT (HFAC + stimulation was delivered 5 min following the initiation of the OGTT, green arrow in Figure 9B). The AUC during the of the HFAC + stimulation OGTTs were constant, no significant differences, but there was a significant decrease in AU compared to sham (Figure 9B, sham = 6228 ± 1293 AU, HFAC + stimulation = 2225 ± 825 AU, *p* = 0.015). We also observed a sustained decrease in PG following cessation of HFAC + stimulation. The average Fasting Plasma Glucose (FPG) for the sham group was 120 ± 14 mg/dL which was consistent over 3 OGTTs with 2 days rest between OGTTs. Following the first HFAC + stimulation PG declined to 67 ± 5 mg/dL. Interestingly, the FPG remained decreased at 69 ± 4 mg/dL at the 3rd HFAC + stimulation OGTT experiment. To determine if HFAC + stimulation applications maintained a decrease in FPG, 3 additional HFAC + stimulation experiments (3 h 55 min application) were conducted (2 days separation between HFAC + stimulation applications, Figure 4). The depression of FPG compared to sham FPG was consistent, meaning there was not a steady decrease in FPG over time. At 16 days following the first HFAC + stimulation experiment the average FPG was 71 ± 4 mg/dL (comparison for all time points consisted of a One-Way ANOVA test with *p* = 0.045 and *F*-statistic = 3.47). A control washout OGTT (with no application of HFAC + stimulation) after HFAC + stimulation experiments demonstrated the OGTT AUC returned/recovered to sham conditions, albeit slightly attenuated but not significantly different (4393 ± 2835, *n* = 3).

**FIGURE 10:**
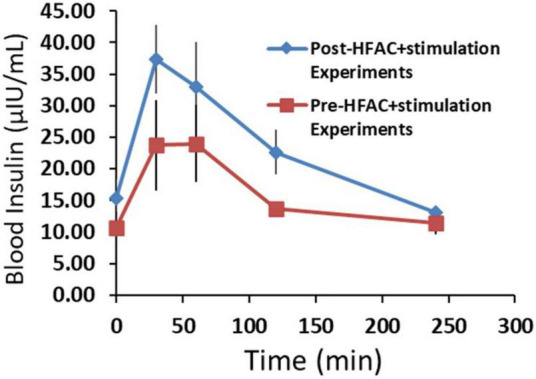
The insulin response was not attenuated following six applications of high frequency alternating current (HFAC) + stimulation (Post-HFAC + stimulation Experiments) vs. Sham (Pre-HFAC + stimulation Experiments). *n* = 3 swine Error bars are SEM.

To gain insight on beta cell function following applications of HFAC + stimulation, insulin was measured during an OGTT prior to and following all HFAC + stimulation experiments (6 experiments total *n* = 3 swine) in the Alloxan treated swine. There was a slight, but non-significant, increase in baseline insulin following HFAC + stimulation experiments (Baseline prior to HFAC + stimulation = 10.7 ± 2.6 μIU/ml vs. baseline following HFAC + stimulation tests = 15.4 ± 2.9 μIU/ml, *p* = 0.28). Insulin increased during the OGTT to an apparent larger, but non-significant, degree following the HFAC + stimulation experiments (Figure 10). This suggested that following multiple HFAC + stimulation procedures beta cells ability to release insulin was not affected. This demonstrated the durability of pancreatic function (in the context of insulin release) with HFAC + stimulation in this set of experiments.

Our rat results, and numerous studies (Daniel and Henderson, 1967; Frohman et al., 1967; Kaneto et al., 1967; Adrian et al., 1983; Berthoud et al., 1983; Ahren and Taborsky, 1986), have demonstrated that stimulation of Vagus nerve fibers innervating the pancreas does not induce a decrease in glucose. However, there are mixed reports about whether hepatic vagotomy decreases glucose (Okazaki et al., 1993; Bernal-Mizrachi et al., 2007). To address this, we tested if HFAC to the hepatic branch, would decrease glucose following an OGTT. We found that there was no difference in AUC with HFAC (AUC = 5364 ± 879 AU, *n* = 3) compared to sham OGTTs. These results were similar to the results from the hepatic vagotomy treatment in the ZDF rat experiments.

Using the same experimental design, similar and significant glycemic control was observed in 3 non-Alloxan treated control swine (FPG = 68 ± 3 mg/dL) with HFAC + stimulation. An OGTT prior to and following implantation of the devices demonstrated no significant difference in glycemic control due to device implant (pre-implant AUC = 1582 ± 451, post-device initiation AUC = 1164 ± 172 *p* = 0.41). However, following the initiation of the HFAC + stimulation OGTT there was a significant decrease in glucose compared to pre-device initiation (HFAC + stimulation AUC = 583 ± 131, *p* < 0.05, Student’s test).

### Nerve histopathology

**FIGURE 11:**
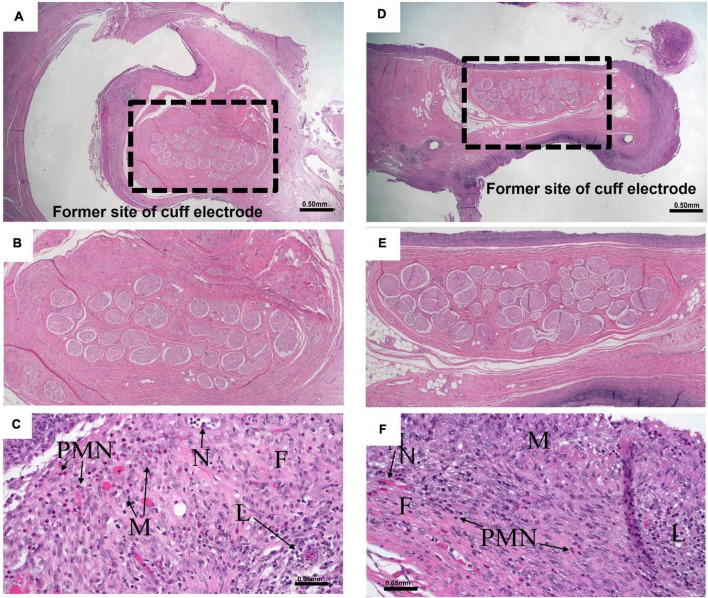
Nerves and surrounding tissue were healthy after 5 of high frequency alternating current (HFAC) + stimulation applications. **(A)** A 10× view of the Vagus nerve surrounded by space formally occupied by electrode delivering HFAC. Scale bar 0.5 mm **(B)** Expanded view of nerve facials taken from the dashed box in panel **(A)**. **(C)** Normal nerve surrounded by a minimally thick fibrous capsule with moderate fibroblasts and 5–10 vascular profiles present per field of view at 200× total magnification that were fine new blood vessels. There was minimal subacute hemorrhage in the capsule and mild amounts of necrosis along the margin of the capsule. Moderate numbers of lymphocytes and macrophages and mild numbers of polymorphonuclear cells infiltrated the capsule. Scale bar 0.05 mm. Abbreviations: F, fibrous capsule; N, neovascularization; L, lymphocytes; PMN, polymorphonuclear cells; M, macrophages. **(D)** A 10× view of the Vagus nerve surrounded by space formally occupied by electrode delivering stimulation. Scale bar 0.5 mm. **(E)** Expanded view of nerve facials taken from the dashed box in panel **(D)**. **(F)** The slide consisted of normal nerve surrounded by a minimally thick fibrous capsule with mild fibroblasts and 5–10 vascular profiles present per field view at 200× total magnification that were fine new blood vessels. There was mild subacute hemorrhage in the capsule. Minimal numbers of polymorphonuclear cells, moderate numbers of lymphocytes, moderate numbers of macrophages, and minimal numbers of multinucleated giant cells infiltrated the capsule. Scale bar 0.05 mm. Letters correspond to the exact abbreviations as in panel **(C)**.

Histopathology was conducted following HFAC + stimulation experiments to access nerve health. All histopathology findings were expected and interpreted to be of no major clinical significance. All Vagus nerve fibers and constituent axons within the cuff void were morphologically normal. There was no evidence of tissue damage at the cuffed electrode contact with the Vagus nerve. For all nerves the epineurium thickness range was estimated to be from 0.05 to 0.36 mm. The host reaction to the cuff sheath and surgical fixation sites were typical for the animal model at the post-implantation time point (example nerve section for HFAC and stimulation sites in Figures 11A–F).

## Discussion

This study tested if a targeted dual bio-electronic technique of high frequency alternating current (HFAC @ 5,000 Hz) applied to the hepatic branch of the Vagus nerve with simultaneous concurrent low frequency stimulation (1 Hz) of the celiac branch of the Vagus nerve (HFAC + stimulation) will improve glycemic control. This was tested acutely in Sprague Dawley and T2DM ZDF rats as well as in glucose intolerant Alloxan treated Yucatan swine. In all of the animal models tested there was consistent HFAC + stimulation-induced improvement of glycemic control, as measured by AUC, following a glucose challenge as well as a decrease in FPG in Alloxan treated swine.

The animal model systems utilized in this study consisted of a well-established T2DM ZDF rat model (Friedman et al., 1991; Sparks et al., 1998; Corsetti et al., 2000; Schmidt et al., 2003; Reinwald et al., 2009) and glucose intolerant Alloxan treated porcine (glucose intolerance demonstrated in Figure 8). Typical generation of diabetic swine uses Alloxan at doses of 100–125 mg/kg (Boullion et al., 2003) which will cause insulin dependence due to a high level of beta-cells ablation; more characteristic of a T1DM model. Sinclair Bio Resources (Auxvasse, MO, USA) has created a proprietary method of developing non-insulin dependent, glucose intolerant, Yucatan swine. This method uses a lower titrated dose of Alloxan which induces a partial ablation of beta-cells. With a high fat/caloric diet this technique produces insulin resistance and glucose intolerance without insulin dependency; suggestive of a T2DM model. Also, there was an decrease in FPG between control swine that did not receive Alloxan and the Alloxan treated swine. Control FPG was 68 ± 3 mg/dL compared to 120 ± 14 mg/dL in Alloxan treated swine.

Isolated Vagus nerve electrophysiology suggested that the application of 5,000 Hz HFAC created a reversible conduction block in the *in vivo* studies. In the isolated nerve experiments, there was no significant change in the CAP elicited by the proximal control stimulation electrode at 8 mA following HFAC (Figure 7B). In contrast there was a complete disappearance of the CAP elicited by the distal stimulation electrode at this amperage, suggesting a localized conduction block at site of the HFAC electrode (Figure 7B). The application of 5,000 Hz has also been shown to induce a reversible blockade in sub-diaphragmatic rat Vagus nerves (Waataja et al., 2011) and the carry over block has been seen in single fiber recordings following the application of HFAC *in vivo* (Cuellar et al., 2012). Parameters to induce block in the isolated Vagus nerve electrophysiology experiments were likely to translate to parameters required to induce conduction block *in vivo* due to the use of similar electrodes with similar electrode nerve contact, spacing between electrodes and similar impedances (∼1000 Ohms). There was a washout effect in the Alloxan treated swine study with the devices turned off, suggesting reversibility of the HFAC + stimulation procedure.

Evidence from our prior studies, and those found in this study, suggest that the HFAC and stimulation parameters used in the rat experiments induced a reversible conduction block and nerve excitation, respectively. We have previously demonstrated, with the same electrodes and HFAC signal as in this study, a conduction block of Aδ- and C-fibers in isolated sub-diaphragmatic rat Vagus nerve (Waataja et al., 2011). Supporting evidence in the present study was demonstrated in the control and ZDF rat experiments where performance on the IVGTT with vagotomy + stimulation mirrored that of HFAC + stimulation, but not that of stimulation alone (Figure 6). Reversibility was demonstrated by a subsequent IVGTT in the ZDF rat study, with the HFAC and stimulation turned off, yielding a similar result as the sham group. The negative monophasic stimulation pulse width and amplitude used in the rat experiments are within the parameters used for nerve excitation in our previous isolated sub-diaphragmatic Vagus rat nerve study (Waataja et al., 2011).

Interestingly, following 2 weeks of applications of HFAC + stimulation in the porcine study, FPG remained decreased compared to pre-device initiation FPG levels. The mechanism of the persistent decrease in baseline glucose was beyond the scope of this study, however, other clinical and preclinical studies investigating HFAC-induced conduction block or stimulation of peripheral nerve have demonstrated long lasting physiological and cellular changes lasting hours to weeks following cessation of the signals (Liu and Sandkuhler, 1995; Sandkuhler, 2007; Biggio et al., 2009; Soin et al., 2015). In a study by Soin et al. (2015) 5,000 Hz was applied for 30 min to the sciatic nerve of amputees to study pain relief. It was found that following the cessation of the 5,000 Hz signal pain relief lasted for up to 9 h. It was discussed that “This extended pain relief duration could be the consequence of ‘winding down’ or desensitization in both the peripheral and central nervous system during the temporary suppression of the afferent signals from the peripheral pain source.” In our case, with 4-h of HFAC + stimulation, neuronal plasticity in vagal nuclei of the brainstem is a possible explanation of our study outcomes. It has been reported that synaptic strength between vagal afferents and their targets within the nucleus tractus solitarius (NTS) are modified by electrical modulation of the Vagus nerve (Zhou et al., 1997; Bantikyan et al., 2009). The electrical signals delivered in this study may have impacted the synaptic strength between vagal afferents and their targets in the brain stem or circuitry within CNS vagal nuclei (i.e., NTS, dorsal motor nucleus of the Vagus and/or nucleus ambiguous) to induce long lasting changes in fasting glucose. Long lasting changes in the hepato/celiac autonomic relay arc induced insulin response (Lee and Miller, 1985) may also have been impacted. Another possibility is that the stimulation of celiac fibers regenerated β-cells. It has been shown that the Vagus nerve controls pancreatic β-cell proliferation (Lausier et al., 2010) and the Vagus nerve has been shown to exert trophic control over other visceral organs such as the stomach (Hakanson et al., 1984). Despite the mechanism, this finding may increase HFAC + stimulation’s therapeutic effect. There were no signs of hypoglycemia (FPG < 50 mg/dL) in our experiments.

It should be noted that there have been observations that Alloxan’s effects can decrease over time in certain animal models (Ighodaro et al., 2017), however, this apparently was not the case in our experiments. Following recovery from IVGTTs PG was 113 ± 9 mg/dL which was similar to sham FPG of 120 ± 14 mg/dL; approximately 10 weeks had elapsed between the two blood samples. Within 3 days between Sham experiments and the conclusion of the HFAC + stimulation experiment PG decreased from 120 ± 14 to 67 ± 5 mg/dL. Following this, plasma glucose levels stayed constant for 2 weeks between the conclusion of the HFAC + stimulation (67 ± 5 mg/dL) and the last FPG measurement (71 ± 4 mg/dL). The simplest explanation from this observation was that the HFAC + stimulation contributed to the sustained decrease in PG.

Anesthesia can affect glucose concentration. It has been shown, for example, that ketamine and isoflurane will induce hyperglycemia (Behdad et al., 2014; Chen et al., 2015). However, sodium pentobarbital has been shown to have little to no effect on glucose in rat (Johansen et al., 1994; Guarino et al., 2013). This anesthesia was used in the rat experiments and there was no change in glucose concentration in the sham group for the 1 h period prior to the IVGTT. In the swine studies there was a 10-day period following surgery before OGTT experiments were conducted. Pre- and post-implant FPG and OGTTs were similar. Pre-device initiation (or sham) OGTTs yielded similar responses over a 1-week period and there was no significant change in FPG during this week. It is unlikely that anesthesia had a significant impact on the results of this study.

There is mixed evidence that hepatic Vagus branch vagotomy may offer increased glycemic control. Hepatic vagotomy may decrease PG by disinhibition of vagal efferent fibers innervating the pancreas (Lee and Miller, 1985), and/or decreased insulin resistance. This was demonstrated in a study by Bernal-Mizrachi et al. (2007). In this study they demonstrated that hepatic vagotomy attenuated hyperinsulinemia, hyperglycemia and insulin resistance through decreased expression of hepatic PPARα (Bernal-Mizrachi et al., 2007). However, there are inherent problems with nerve ligation. Hepatic vagotomy is non-reversible, there may be adaptation to the vagotomy with time and peripheral nerves can regrow (Fawcett and Keynes, 1990) with unknown effects on the newly re-innervated end organ. Indeed, autonomic denervation clinical studies for the treatment of hypertension have failed (Bhatt et al., 2014). Hepatic vagotomy may also cause negative changes in feeding behavior, increased hypoglycemic episodes, may affect liver regeneration and cause increased metastasis during liver cancer (Kiba, 2002; Donovan and Watts, 2014; Hiramoto et al., 2017). In our rat studies, ligation of the hepatic branch did not increase glycemic control following the IVGTT (Figure 6A).

Many studies using electrical stimulation of an intact single Vagus nerve trunk, or branch, have failed to increase glycemic control (Daniel and Henderson, 1967; Frohman et al., 1967; Kaneto et al., 1967; Adrian et al., 1983; Berthoud et al., 1983; Ahren and Taborsky, 1986; Meyers et al., 2016), with few demonstrating an increase in glycemic control (Yin et al., 2019). Vagal stimulation of celiac fibers, or Vagus nerve segments central to the celiac branching point, may have problems due to its effects of pancreatic glucagon release (Adrian et al., 1983; Berthoud et al., 1983; Ahren and Taborsky, 1986). By simultaneously blocking conduction through the hepatic branch we hypothesize that this attenuates the livers sensitivity to glucagon (Xue et al., 2000). In our T2DM rats there was no increase in glycemic control with celiac stimulation (Figure 6A).

It should be noted that in the context of significant and sustained weight loss standalone Vagus nerve stimulation or blockade has demonstrated increased glycemic control (Shikora et al., 2016; Malbert et al., 2017). Weight loss was not a factor in our study; the rat experiments were acute and the swine maintained their weight throughout the study.

Optogenetic and chemogenetic vagal manipulation currently under investigation (Ngo et al., 2020; Okonogi and Sasaki, 2021) offer impressive neuronal specificity and may impact glycemic control, however, viral vector genetic modulation presents a clinical risk and is currently clinically unrealistic. Electrical vagal neuronal stimulation has a proven clinical safety and efficacy profile for other indications (Gonzalez et al., 2019) and may be a practical method of vagal modulation for glycemic control.

The pulse widths (∼4 ms) and current amplitudes (∼8 mA) applied in these experiments were relatively high compared to other Vagus nerve stimulation parameters used clinically (Heck et al., 2002) in which stimulation is applied to the cervical Vagus nerve. It is apparent that excitation of non-myelinated C-fibers of the sub-diaphragmatic Vagus nerve require significantly more energy to excite than the fiber types stimulated in cervical Vagus nerve stimulation. In our previous rat Vagus nerve electrophysiology studies in rat (Waataja et al., 2011) pulse widths and current amplitudes up to of 4 msec and 8 mA, respectively, were required to excite C-fibers of the sub-diaphragmatic Vagus nerve. In our current study, pulse widths of 4 ms and current amplitudes of 8 mA were required to elicit C-wave CAPs in isolated swine Vagus nerve. Histopathology demonstrated healthy nerve (Figure 11) following stimulation in this study.

It should be noted that in contrast to the *in vivo* swine experiments there was no change in PG during the treatment period in the rat experiments prior to the IVGTT for HFAC + stimulation. The shorter treatment time in the acute rat experiments compared to swine experiments may explain the different findings. The longer duration swine study would most resemble the conditions under human use.

There is great need for T2DM treatments due to growth rate, increased risks of comorbidities and cost to the healthcare system. With current growth trends, the diabetic population will increase to 366 million people worldwide by 2030 (Brunton, 2008). Western nations will be seriously affected; by 2050, it has been estimated as many as 1 in 3 US citizens will be diabetic (Boyle et al., 2010). Progression of T2DM significantly increases risks of stroke, myocardial infarction, microvascular events, and mortality (Stratton et al., 2000). Diabetics face average medical expenditures directly attributable to the disease of $9,600/year (American Diabetes Association, 2018).

In conclusion, we have discovered a novel targeted dual Vagus nerve bio-electronic modulation technique for enhancing glycemic control. To the best of our knowledge, this is the first study that used different patterns of electrical signals sent to two *separate* sub-diaphragmatic Vagus nerve branches to induce a therapeutic effect; in this case a possible treatment for T2DM. This was demonstrated in experiments utilizing the well-established Zucker rat model of T2DM and suggested in experiments using glucose intolerant Alloxan treated swine. The standalone techniques of applying HFAC to the Vagus nerve and Vagus nerve stimulation, have been shown to be safe in humans and these techniques are approved for obesity, epilepsy, and depression (Shikora et al., 2016; Gonzalez et al., 2019). This suggests a promising safety profile with the techniques used in this study. Since HFAC + stimulation increased glycemic control 5 min following offering the swine glucose gives inspiration for HFAC + stimulation to be initiated at the start of a glucose spike by working with continuous glucose monitoring technology to form a closed loop system to blunt glucose spikes on demand. Furthermore, the system would have the ability to work with AI and machine learning to optimize HFAC + stimulation parameters over time.

## Data availability statement

The datasets presented in this article are not readily available because there are datasets in this manuscript that are proprietary. Requests to access the datasets should be directed to JW, jwaataja@reshapelifesci.com.

## Ethics statement

The animal study was reviewed and approved by Institutional Animal Care and Use Committee at North American Science Associates, Inc. (Brooklyn Park, MN, United States) as well as the Institutional Animal Care and Use Committee at the University of Minnesota.

## Author contributions

JW and RN performed rat and porcine glycemic control experiments. CH performed electrophysiology experiments. CB lead all experiments. All authors contributed to the article and approved the submitted version.
